# A von-Neumann-like photonic processor and its application in studying quantum signature of chaos

**DOI:** 10.1038/s41377-024-01413-5

**Published:** 2024-03-14

**Authors:** Shang Yu, Wei Liu, Si-Jing Tao, Zhi-Peng Li, Yi-Tao Wang, Zhi-Peng Zhong, Raj B. Patel, Yu Meng, Yuan-Ze Yang, Zhao-An Wang, Nai-Jie Guo, Xiao-Dong Zeng, Zhe Chen, Liang Xu, Ning Zhang, Xiao Liu, Mu Yang, Wen-Hao Zhang, Zong-Quan Zhou, Jin-Shi Xu, Jian-Shun Tang, Yong-Jian Han, Chuan-Feng Li, Guang-Can Guo

**Affiliations:** 1 CAS Key Laboratory of Quantum Information, University of Science and Technology of China, Hefei, 230026 China; 2 CAS Center for Excellence in Quantum Information and Quantum Physics, University of Science and Technology of China, Hefei, 230026 China; 3 Hefei National Laboratory, University of Science and Technology of China, Hefei, 230088 China; 4 Research Center for Quantum Sensing, Zhejiang Lab, Hangzhou, 310000 China; 5 Quantum Optics and Laser Science, Blackett Laboratory, Imperial College London, Prince Consort Rd, London, SW7 2AZ UK; 6 Clarendon Laboratory, Department of Physics, Oxford University, Parks Road OX1 3PU, Oxford, UK

**Keywords:** Quantum optics, Quantum optics

## Abstract

Photonic quantum computation plays an important role and offers unique advantages. Two decades after the milestone work of Knill-Laflamme-Milburn, various architectures of photonic processors have been proposed, and quantum advantage over classical computers has also been demonstrated. It is now the opportune time to apply this technology to real-world applications. However, at current technology level, this aim is restricted by either programmability in bulk optics or loss in integrated optics for the existing architectures of processors, for which the resource cost is also a problem. Here we present a von-Neumann-like architecture based on temporal-mode encoding and looped structure on table, which is capable of multimode-universal programmability, resource-efficiency, phase-stability and software-scalability. In order to illustrate these merits, we execute two different programs with varying resource requirements on the same processor, to investigate quantum signature of chaos from two aspects: the signature behaviors exhibited in phase space (13 modes), and the Fermi golden rule which has not been experimentally studied in quantitative way before (26 modes). The maximal program contains an optical interferometer network with 1694 freely-adjustable phases. Considering current state-of-the-art, our architecture stands as the most promising candidate for real-world applications.

## Introduction

Photonic system plays important role in quantum simulation^1–6^ and quantum computation^7–35^ and other applications such as machine learning^36,37^. It has the merits of high coherence, robustness in ambient temperature and pressure environment. Besides, it is also very convenient for information transmission, which is essential for quantum network and distributed computation^38^.

The milestone of universal quantum computation with linear optics is the Knill-Laflamme-Milburn (KLM) scheme^7^, before which, people believe that non-linear effect (e.g., Kerr non-linearity) is indispensable to provide the photon-photon interaction^8^. KLM utilize the measurement-related “hidden non-linearity”^7^ and Hong-Ou-Mandel (HOM) effect (photon indistinguishability induced exchange interaction) to propose a viable way to realize universal quantum computation based on linear optics. One essential gate in KLM scheme is the non-linear sign change (NS) gate. This gate is, however, non-deterministic, which has a largest success probability of 1/4, as has been proved in both numerical^9^ and analytical^10,11^ ways. Thus, the controlled-phase (CZ) gate, which can be constructed by two independent NS gates and the HOM effect, is also non-deterministic. There are several ways to make a (near-)deterministic CZ gate. One way is to utilize entanglement, teleportation, measurements and feedforward to realize CZ gate with success probability of *n*^2^/(*n* + 1)^2^ (*n* is the qubit number in the ancillary entanglement state)^7,8^. Another way is to utilize a memory with single-photon emissions, measurements and repeated operations to realize CZ gate deterministically^12^. Besides the KLM scheme, a similar computation model based on cluster (entangled) state and measurement are proposed, also known as one-way quantum computation^13–15^. These universal models cost a lot of resources, e.g., entanglement and number of measurement operations, and still very hard at current state-of-the-art.

Nowadays, a great interest is attracted in a game called Boson sampling^16^ and its variants, i.e., Scattershot Boson Sampling^17^ and Gaussian Boson Sampling (GBS)^18^. These games describe that a multi-photon state goes through a multimode unitary evolution and the final photon-number distribution at each mode is required. They are non-universal computation models, but can demonstrate the quantum advantage over classical computer with the size being large enough. After the efforts during a decade^19–26^, quantum advantage based on GBS has been demonstrated^22,25^. Especially fortunately, GBS is recently found to have connection with graph theory^27–29^ and can be used to solve quite a lot of hard problems in molecular dynamics^30,31^ and other fields like medicine, etc. This makes GBS a powerful tool in solving many hard real-world problems which cannot be efficiently solved in classical computer, but not only a quantum game.

As a quantum processor that is suitable for real-world applications, the universal programmability is the basic requirement; even if in current non-universal computation model of GBS, the universal programmability of the multimode-unitary-evolution circuit is necessary^21,26,32–34^. To be distinguished, we call the later multimode-universal (MM-universal) programmability (we note that in some previous works, people also would like to just simply call it “universal”^21,26^). This requirement will make the processor meet various real-world conditions. Our work just focuses on the MM-universal programmability of the photonic processor.

MM-universal programmability is another critical ability for photonic processor that is as important as quantum advantage, but these two abilities have an approximate tradeoff relation, in which the loss plays a role. To date, some experiments give up or partially sacrifice the MM-universal programmability to reduce loss thus achieve quantum advantage^22,25^, and some others insist on the way of MM-universal programmability but the loss is difficult to be reduced^21,32,33^, and the largest size of MM-universal photonic processor is 20 modes with 380 phase shifts^33^ which is not enough to show quantum advantage. Besides the degree of programmability, different systems also affect the loss, and generally speaking, on-table optics (or bulk optics) will have less loss than integrated optics. We can see that the quantum-advantage-demonstration experiments are all performed on-table currently. Moreover, most current photonic processors are in unlooped configuration. This feature is not friendly to MM-universal programmability, which needs a huge number of free real parameters, i.e., phase shifts. These phase shifts will cost a great of resources, e.g., phase shifters, electric-pulse generators and the rooms for installing them, etc., and make the experiment both expensive and cumbersome. This shortcoming is less obvious in integrated optics because of the small size of photonic chip (but the electric-pulse generators and cables are still complex, besides, the heating effect and the crosstalk among different electrodes by heat will be a problem), so we see that the experimental demonstrations of MM-universal programmability are all in integrated optics. Therefore, at current stage of technology level, the potential of current photonic processors to be used in real-world applications to surpass classical computer is restricted.

Here we describe another architecture for photonic processors which has a looped structure and encodes on temporal mode of photons (temporal-mode encoding is also feasible for the photon circulation compared to spatial mode). This architecture enables the construction of a MM-universally programmable circuit with resource-efficiency and software-scalability. The on-table optical system enables this architecture with relatively less loss and the potential to achieve quantum advantage in near future based on current technology level (see Supplementary Information of ref. ^35^). This architecture will break the restrictions mentioned above and make photonic processors promising in the real-world applications, and actually the second-generation of processor following this architecture has been used for molecular docking and RNA folding^35^.

## Results

As mentioned previously, we present an architecture for photonic processors and focus on the MM-universal programmability of the circuit. The looped structure requires that this architecture should include a quantum memory to temporally store the photon qudit. This makes this architecture seem like the well-known von-Neumann/Harvard (VH) architecture^39^. Both these two architectures store data and program instructions in memories, and fetch them one step by one step for data processings. The difference is that the data and program in von-Neumann architecture are stored in the same memory but those in Harvard architecture are in different memories. Nowadays, these two original architectures are not often used, but instead by more complicated hybrid architectures. Nevertheless, people still generally called them von-Neumann architecture^40^, and as our model is more like Harvard architecture, we simply call it the VH architecture.

**Fig. 1:**
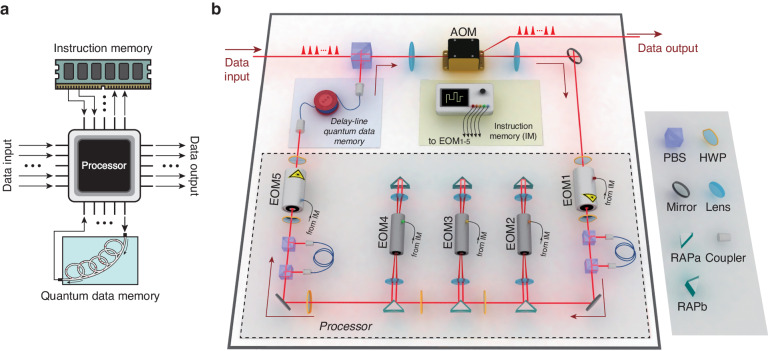
**Hardware of the VH architecture photonic processor utilizing temporal-mode encoding**. **a** A typical structure of quantum VH architecture. It comprises separated processor unit, instruction- and quantum-data-memory units. The data after input, are repeatedly exchanged between the processor and data-memory units to be processed according to the instructions or temporarily stored, until they are output for detection. **b** Brief sketch of the hardware. Although the practical hardware construction is much more complicated, this sketch can comprehend the essence of this system. Compared to the figure a, EOM2-4 with continuously-variable phase shift universally simulate quantum gates according to instructions, EOM1 and EOM5 with two-valued phase shift play the role of address control also following the instructions, and they constitute the processor unit; the whole light path includes a big circulation (benefited by the utilization of temporal mode) with the data repeatedly and alternately going through the processor unit and a delay-line quantum-data-memory unit (similar role as that in EDVAC and UNIVAC I), which corresponds to the frequent data exchange between processor and data memory; an instruction memory unit store the programs and accordingly generate electrical signals; the pulses before the entrance PBS represent the data input with temporal mode, and pulses after AOM is data output. The phase stability analysis can be found in Supplementary Information

A typical VH architecture, precisely, the Harvard architecture, is sketched Fig. 1a. After the data are input into the processor, the processor will acquire instruction from the instruction memory, and then it prepares an operation according to the instruction to handle the data. At the end of the single loop, the processed data are sent into the quantum data memory. In the next loops, the data are repeatedly acquired from and buffered into the data memory, and simultaneously the corresponding instructions are read out from the instruction memory to guide the processor to prepare the suitable gates. Until the task is finished, the final data are output for detection. We also see the superconducting quantum processor with von-Neumann architecture^40^, but it does not affect the advantage of our work in photonic system. The reason is as following. In the matter systems^40–50^, the data carriers can play as the memories by themselves, and the in-memory-computing architecture^51,52^ is more preferred. In contrast, von-Neumann architecture containing frequent data exchange between memory and processor is harmful by inducing more data loss and power consumption. However, the situation is completely different in the photonic system, since the photon flies quickly and cannot play as the memory by itself. To construct a looped structure and make the photonic processor resource-efficient, we nevertheless need a memory, i.e., the VH architecture in this case indeed will be helpful.

In our architecture, the temporal modes are used to encode data, and the primary structure used to process the modes is sketched in Fig. 1b. Comparing with the VH architecture in Fig. 1a, the component from EOM1 (electro-optic modulator) to EOM5 is recognized as the processor unit. EOM1 and EOM5 are controlled by digital signals generated by the instruction memory unit to address which data will be handled or not. EOM2-4 are controlled by analog signals also generated from the instruction memory unit to create an arbitrary unitary gate. The processed data can be temporarily sent into the delay-line quantum-data-memory unit (the same as that in the early-age classical computers, such as EDVAC and UNIVAC I^53,54^), and they will be retrieved for the next instructed operations in the following loops. The pulses before the entrance PBS (polarizing beam splitter) and those after AOM (acousto-optic modulator) represent the data input and output, respectively, in temporal mode. The software of this processor is a series of instructions written in the instruction memory unit in advance. To fulfill a task, it should be compiled into an instruction series first. An example of the complicated instruction series will be found in the Supplementary Information. The temporal mode is more stable in phase, and most suitable for the loop-structure construction and also for long-distance transmission, since it is insensitive to the slow phase shifts (induced by mechanical shaking or temperature drift) and the spatially nonuniform environmental noises (including photon losses caused by mirror imperfections or coupling-efficiency difference). The temporal-mode encoding and circulation structure in VH architecture make this processor can be resource-efficiently scalable in both mode number and evolving depth just by using software.

To illustrate the unique capacities of our photonic VH-architecture processor, we utilize it to investigate the quantum signature of chaos, which is a widely interested fundamental problem^55–67^ and is closely related to the classical-quantum boundary^56,57,60^, and can also be used to benchmark quantum simulators^64,65^. Quantum signature of chaos is investigated in this work from two different aspects which can be compiled into two quantum programs. These two quantum programs are able to be executed on the same photonic processor, respectively (in a resource-efficient way), though they require different resources, i.e., numbers of temporal modes and evolving steps. The first program investigates the wavefunction distributions in phase space, which provide visual pictures to show the different behaviors in regular and chaotic regions. The second program, requiring more resources, quantitatively investigates the Fermi golden rule (FGR) of the quantum signature of chaos^66,67^, which is derived from the random matrix theory (RMT)^61–63^ and was never quantitatively confirmed in experiment before.

We begin with the quantum kicked-top model (QKM), whose classical counterpart is usually employed to study classical chaos. The first quantum task is to simulate the evolution of this model and give a visual picture of the evolved states in both regular and chaotic regions. This model is governed by a periodic Hamiltonian

1 $$H(t)=(\hslash {p}/\tau ){J}_{y}+(\hslash k/2j){J}_{z}^{2}{\mathop{\sum} \limits_{n=-\infty }^{+\infty }}\delta (t-n\tau )$$

where $J$ is the angular momentum with ${J}^{2}=j(j+1)$ ($j$ is chosen to 6 in our experiment); and ${J}_{y}$ as well as ${J}_{z}$ are the corresponding angular momentum operators, whose explicit expressions can be found in the Supplementary Information. $p$ represents the rotation angle about $y$ axis, the $\delta$ function represents the kicks and $k$ is the kick strength determining the regular or chaotic degree of the dynamics, $\tau$ represents the duration between kicks. This system has an equivalent Floquet representation

2 $$U={{\rm{e}}}^{-i\pi {J}_{y}/2}{{\rm{e}}}^{-{ik}{J}_{z}^{2}/j}$$

**Fig. 2:**
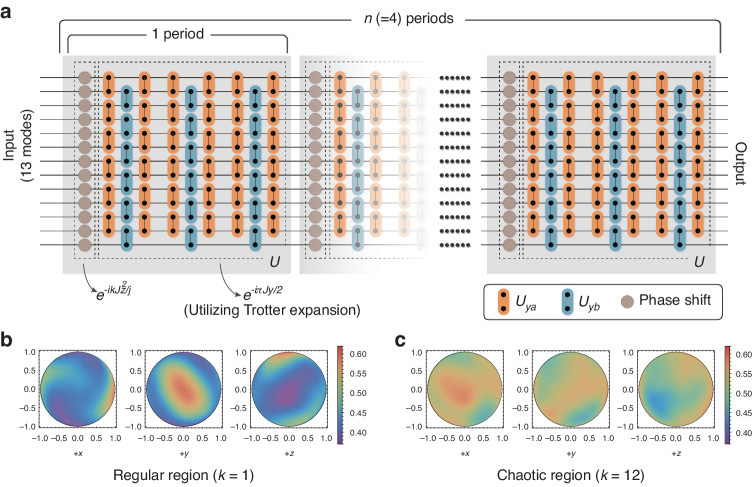
**The first program to distinguish regular and chaotic behaviors**. **a** Logic diagram. 13 temporal modes are created in program and each layer in this figure represents one loop in Fig. 1b. Pairs of modes are controlled to be coupled and performed with two-level operations according to instructions. These operations are represented by circles with two dots, and those without dots stand for the phase gates. For the quantum kicked-top model utilized here, each period of evolution $U$ comprises 9 layers of two-level operations based on Trotter expansion and 1 layer of phase gate, and totally $n=4$ periods are compiled in this program. **b**, **c** Running results with parameters $k=1$ and $k=12$, respectively, in the form of Husimi distribution (phase space). Compared to (**c**), the chaotic region, (**b**) corresponding to regular region is less dispersive

To compile the quantum task into a series of instructions (a program), we first decompose the subevolution ${{\rm{e}}}^{-i\pi {J}_{y}/2}$ of $U$ into some simple matrices using Trotter expansion (see Methods), and then further translate $U$ into an instruction string (see Supplementary Information). The instruction string is called a period, in which the program creates a buffer of $2j+1=13$ temporal modes, as shown in Fig. 2a. Our whole program iterates the instruction string 4 times (repeat $n=4$ periods of $U$).

We use the Husimi distribution $R(\theta ,\phi )$^68^ ($(\theta ,\phi )$ is parameters of a coherence state |*θ,ϕ*〉^69,70^ which located on a spherical surface with fix radius) as the visual picture to distinguish the regular region and the chaotic region in QKM. Husimi distribution is a widely used quasi-probability distribution to study the correspondence between quantum phase space distributions and the classical phase space structure^71^. For any state $\rho$, its Husimi distribution is defined as $R\left(\theta ,\phi \right)=\left\langle \theta ,\phi ,|,\rho ,|,\theta ,\phi \right\rangle$. See details in Supplementary Information.

In this quantum task, the initial state ${\rho }_{0}$ is chosen to be a coherent state (a point in the phase space), and we measure all the states after each evolution period (shown in Fig. 2a), denoted as $\{{\rho }_{i}(0\le i\le n)\}$. In experiments, $s=35$ initial coherent states in the phase space are randomly selected to perform the same evolution to obtain the whole experimental density-matrix set $S=\{{\rho }_{i}^{q}{\rm{|}}i=1,2,\ldots ,n{\rm{;}}q=0,1,\ldots ,s\}$. We then calculate the average Husimi distribution $Q\left(\theta ,\phi \right)$ on $S$ (see Methods). The results for $k=1$ are shown in Fig. 2b (regular region), and for $k=12$ are in Fig. 2c (chaotic region). $+x$, $+y$ and $+z$ represent the different view angles of the spherical surface (more data are found in Supplementary Information). It is clear that when the system is in chaotic region (Fig. 2c), the distribution is much more uniform than that in regular region (Fig. 2b), which means the system states are much more dispersive for chaotic region than that for regular region.

Besides the visually distinguishing of the regular and chaotic regions from aspect of Husimi distribution, our high-fidelity processor (>0.992 for one period) allows us to give a deeper quantitative investigation of FGR in the quantum chaotic region. The FGR can be described by the exponential damping of the average fidelity, which is averaged on different initial states in the whole space, along the evolution periods^66^. For a given initial state, the fidelity is defined between the evolved states going through the evolution $U$ with and without perturbation $P={{\rm{e}}}^{-i\delta {J}_{z}}$ ($\delta$ represents the perturbation strength). The region governed by FGR is called Fermi golden region, which is a subregion of chaotic region, and it notes that the decay rate of the average fidelity is independent of $k$ in this region.

**Fig. 3:**
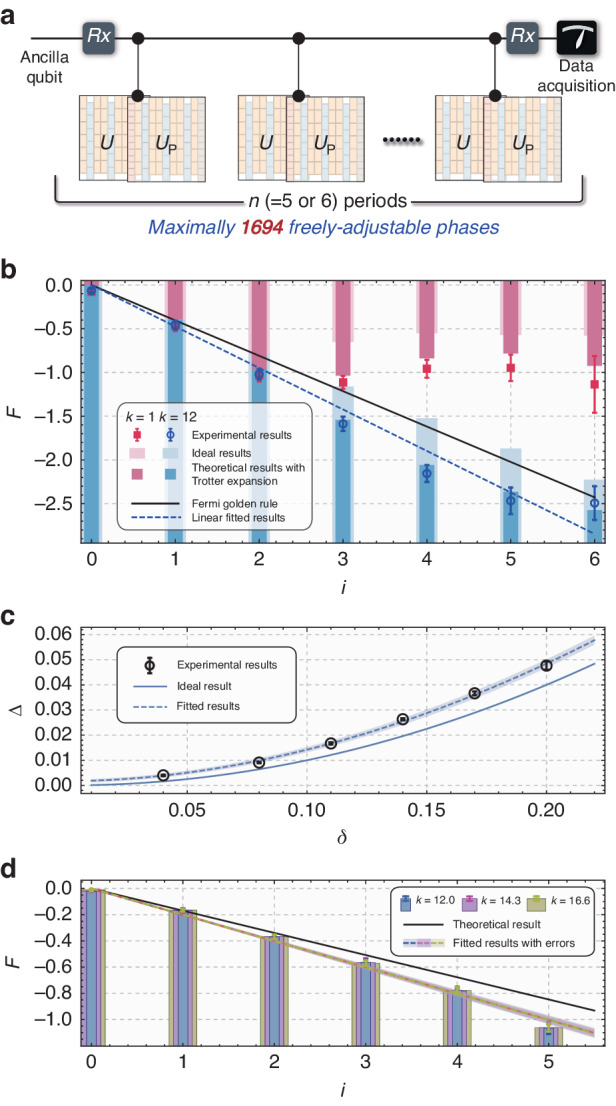
**The second program to quantitatively study FGR**. **a** Brief sketch of logic diagram. In this program, 26 temporal modes are created. 13 of them are still performed with $U$ (i.e., the program in Fig. 2), and the other 13 are performed with ${U}_{P}$. This structure constitutes a big controlled gate by an ancilla qubit. $n$ = 5 or 6 periods of evolutions are compiled in this program. The ${R}_{x}$ operation belongs to the state preparation and detection, and is performed by the transformations between temporal to polarization modes (See Supplementary Information). Using this algorithm, the average fidelity $\bar{F}$ can be directly readout. **b** Fidelity decay under $k=1$ and $k=12$. When $k=1$, fidelity fluctuates as the period index $i$ grows; when $k=12$, fidelity decays following FGR, and the fitted slope of F denoted as $=c(j)\triangle$ is just the decay rate, where $c(j)$ is a negative constant related to $j$. **c** Fidelity decay rates represented by $\triangle$ with various $\delta$ under $k=12$. It demonstrates that the decay rate is proportional to ${\delta }^{2}$ within the errors of Trotter expansion, and the fidelity decay rate coincidences with FGR. **d** shows that when the evolution is located in Fermi golden region, a constant decay rate will be observed under various *k*

The FGR can be explored by the second program on the same processor (an example of the compiled control sequences can be found in Supplementary Information). In this program, besides the 13 modes used to simulate the evolution of $U$ in the first program, another 13 temporal modes are created to simulate the evolution ${U}_{P}={UP}$ with perturbation. Combining these two parts (total 26 modes) will form a controlled evolution: an ancilla qubit controls a 13-dimensional qudit (see Fig. 3a).

According to the algorithm in ref. ^67^, the average on initial states in the whole qudit space can be completed by preparing the initial state as maximally mixed state ${{\rm{I}}}_{13}$, and the average fidelity between quantum states ${U}^{i}{\rm{|}}{\phi }_{0} \rangle$ and ${U}_{P}^{i}{\rm{|}}{\phi }_{0} \rangle$ ($|{\phi }_{0} \rangle$ represents an arbitrary initial state, and $0\le i\le n$ indicates the number of the periods) can be directly obtained through the Hadamard test circuit by detecting the expectation value of the ancilla qubit on ${\sigma }_{z}$ (see Fig. 3a). This average fidelity is written as ${\bar{F}}_{i}=\bar{{|\langle {\phi }_{0},|,{({U}^{\dagger })}^{i}{U}_{P}^{i},|,{\phi }_{0}\rangle|}^{2}}$, and the overline denotes the average on the space.

Figure 3b shows the dynamics of the experimental average fidelity ${\bar{F}}_{i}$ ($0\le i\le 6$) in the cases of $k=1$ (regular region) and $k=12$ (chaotic region). To better illustrate the exponential decay of the average fidelity, we define ${\rm{F}}=\mathrm{ln}\bar{F}$, then F will be linear with the period index $i$ if FGR is obeyed. The black solid line is the theoretical prediction of FGR. The red symbols represent $k=1$ case, and the blue symbols are $k=12$ case. The bars with light colors are the theoretical results with ideal $U$ evolutions, and bars with deep colors are the theoretical results with Trotter-expanded $U$. The circles (squares) with error bars represent the experimental results, and the errors are derived by the standard deviation of 5 repeated experiments (the same in Fig. 3d). We see the Trotter expansion makes the values of F systematically less than their theoretical values, but it does not affect the main conclusions. The experimental results coincide well with the theoretical values with Trotter expansion. For the $k=1$ case, the average fidelity deviates from the exponential decay after $i=3$ and remains around a certain stable value. For the $k=12$ case, we see a good agreement between the experimental results and FGR within the error of Trotter expansion. The blue dashed line is the linear fitting of these data. These values of fidelity can be regarded as the indicator of quantum signature of chaos^66^.

To further quantitatively detect the properties of FGR, we study the relation between the decay rate of average fidelity (slope of F) and the perturbation strength $\delta$. The experimental value of the decay rate, $\triangle$, with different $\delta$ are shown in Fig. 3c as hollow circles, and error bars are fitting errors. These circles are fitted using $f\left(b,a\right)={\delta }^{b}+a$, and the fitted parameters are $b=1.9030\pm 0.0138$ and $a=0.0017\pm 0.0006$. The shadow in this figure represents the fitting error. This result approximately indicates that the degree of chaos is corresponding to ${\delta }^{2}$, coincident with FGR. The solid line below the circles describes the values of ${\triangle =\delta }^{2}$ which are overall less than the experimental data due to the Trotter expansion.

Besides, the relation between the slope of F and the kick strength $k$ is also studied here. We obtained the slope for three different $k$ ($k=12,14.3$ and $16.6$) and shown in Fig. 3d. The bars with errors represent the experimental results, and the dashed lines and shadows are the linear fittings and fitting errors. The solid line is the ideal theoretical value. Within error bars, we see these three results coincide to each other very well, and this $k$-independence phenomenon indeed reveals one of the key features of the quantum signature of chaos in Fermi golden region.

## Discussion

Using this MM-universally programmable photonic processor with VH architecture, we have run two programs (requiring different resources) compiled into a sequence of instructions to comprehensively investigate the quantum signature of chaos in QKM from both the visual and quantitative aspects. Especially, the Fermi golden region of quantum signature of chaos is quantitatively characterized in experiment for the first time. The temporal mode and the looped structure make the processor intrinsically scalable and can be realized by only controlling software. In contrast, for other photonic processors with fixed mode number and evolving depth which are even MM-universally programmable, to run these two kinds of programs on the same processor will induce either inadequacy or wasting of resources. Besides, these quantum tasks on chaos contain a large optical interference network. Different from the GBS experiment which only needs one multimode evolution, the chaos experiment needs to repeat the evolution many times and requires more real parameters, i.e., freely-adjustable phases. Maximally, our chaos experiment includes 6 periods, and each period has 84 mode couplers and 26 phase shifts (see right panel of Fig. S6a). Each mode coupler contains 3 phases (EOM2-4). Considering another 26 phase shifts at the end of the program, we have totally $(3\times 84+26)\times 6+26=1694$ freely-adjustable phases in this optical network. To make this large optical interference network stable is not easy, especially for the on-table optical experiments encoding on path mode. In our experiment, the relative phases among different modes are quite stable owing to the temporal-mode encoding, utilization of double-core fiber (the two short fiber in Fig. 1b) and the fast light speed. Furthermore, our system is a controllable quantum system, thus the states and coherence contained in them are really evolved in both regular and chaotic regions but not only a mathematical calculation. This situation is different from the simulation on classical computer. Ultimately, the algorithm employed for the FGR experiment provides an exponential speedup on the average-fidelity measurement^67^. Without this algorithm, the average-fidelity measurement will cost a lot of resources and the accuracy for average is also limited by the sampling number and sampling uniformity.

The quantum-memory technology is still under rapid development. For example, a free-space multiplexed delay-line photonic memory is reported recently^72^; the quantum memory based on cold Cs atoms with efficiency of ~90% at storage time of ~10 μs is realized^73^; the quantum memory based on solid-state rare-earth crystal with storage time of 1 h is demonstrated^74^; etc. With these achievements, our VH-architecture processor can be further improved to be fully flexible in the future.

The processor also has potential to be fabricated on chip in the future, based on the development of the integrated-optics technologies, especially the integration of fast electro-optic unit and memory unit.

In addition, our photonic processor is ready to perform GBS^18,27–31^ when its input is changed to squeezed states (the indistinguishability of the photons in these states at diffident modes is also better owing to the temporal-mode encoding), which has been demonstrated in our second generation of processor following the same architecture^35^. At current technology level, considering the MM-universal programmability with acceptable resource cost, photon loss and phase stability, our architecture may be the most promising photonic quantum computing architecture to be put into real-world applications.

## Methods

### Discussions of methods to show flexibility of our VH-architecture photonic processors

**Fig. 4:**
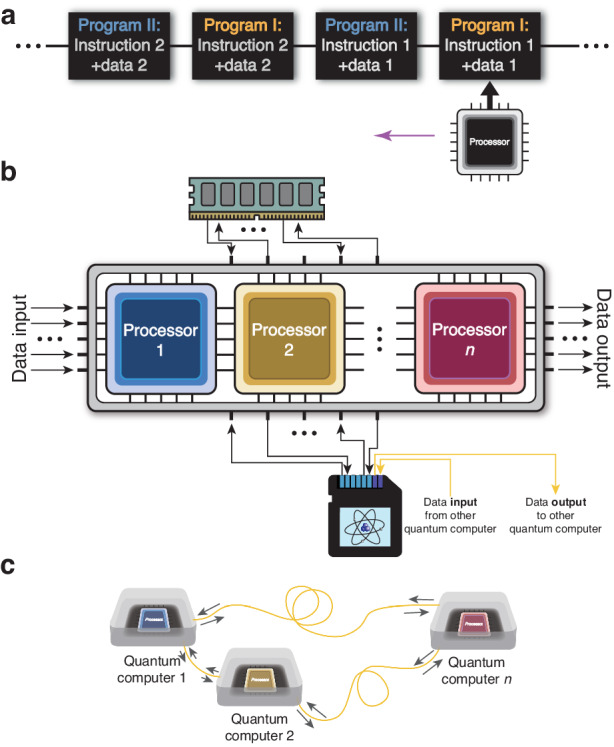
**Flexibility of this photonic VH-architecture processor**. **a** Multiple (time-slice) thread. The programs are divided into series of instruction-data-bindings which are independent of the processor unit. Each binding corresponds to a basic element of these programs. Owing to the memory units in VH architecture, these bindings can be temporarily stored. This makes the binding chains from different programs can be mixed and share the time slices of the processor unit alternately. **b** Multiple core. Also because of the dependence of instruction-data-bindings and the processor, more than one processor cores sharing the memory units can be used to cooperate to fulfill the same task. **c** Distributed computation. When it is extended to a network with each node having an entire set of processor and memories, the data can be transmitted and stored in the memories of the whole net (the yellow arrows in the figure **b**), and all the processors can cooperate to realize a quantum task. During this process, the temporal mode is convenient for phase-stable transmission, and the difficult light-matter interface can be avoided since the information carriers during calculation and transmission are both photons

As that in classical digital computers, the VH-architecture endows our photonic processor with the high flexibility of multiple-thread, multiple-core and distributed computation abilities, as shown in Fig. 4. Although these functions can also be realized in matter-system-based processor without VH-architecture, they will be comparatively difficult for other photonic processors without VH-architecture, and here we focus the photonic system which also has its unique merits compared with matter systems. The primary characteristic of this architecture is that it has independent structures of processor unit, memory units and repeatedly data exchange between them (the circulation), and the whole program can be divided into a chain of instruction-data-binding units which are stored in memory units and not at all related to the processor unit. Therefore, the stored instruction-data-binding units belonging to different programs can be mixed and share the different time slices of the same processor, as shown in Fig. 4a, which we call multiple-thread here. Let us recall the program shown in Fig. 3a which contains a controlled operation. If we ignore the operations on the ancilla qubit, and decompose the controlled operation into two programs respectively corresponding to $U$ and ${U}_{P}$. These two programs can be considered to be executed on two different time threads of the single processor. The time of this processor is equally divided in this example, but in general cases, this can be more flexible with nonuniform and customized time arrangement.

Also because of the separation of processor and instruction-data-bindings, we can use more than one processors to handle the data according to corresponding instruction together, and these processors share the memory units, for which we call multiple-core, as shown in Fig. 4b. These processor cores can be either series-wound or shunt-wound. The former will benefit if the data storage and fetch from memory takes time or has resource losses, and the latter will play roles when the program can be designed to be parallelly executed, as that in classical computer.

Moreover, when we construct a network, with each node including a computer, and the nodes are connected using optical fibers. The data can transmit through the fibers among memories in different nodes (the yellow arrow lines in Fig. 4b), and the processors can cooperate to handle the data in the whole net to fulfill a common task, for which we call distributed computation, as shown in Fig. 4c. The temporal mode used here has the advantage of convenience and phase-stability in transmission among different nodes compared to path mode or other spatial modes, and the photonic coding avoid the light-matter interface between data processing and transmission compared to the matter-based processor in distributed computation.

### Trotter expansion for the subevolution ${{\rm{e}}}^{-i\pi {J}_{y}/2}$

${J}_{y}$ can be decomposed into ${J}_{{ya}}+{J}_{{yb}}$, with ${J}_{{ya}}$ and ${J}_{{yb}}$ having separated blocks of two-level submatrices. The matrix expressions for ${J}_{y}$ and ${J}_{{ya}}$, ${J}_{{yb}}$ can be found in Supplementary Information. Both ${{\rm{e}}}^{-i\pi {J}_{{ya}}/2}$ and ${{\rm{e}}}^{-i\pi {J}_{{yb}}/2}$ can be further decomposed into a series of independent two-level unitary evolutions between each pair of adjacent modes. Since ${J}_{{ya}}$ and ${J}_{{yb}}$ are not commuting, therefore, to realize ${{\rm{e}}}^{-i\pi {J}_{y}/2}$, Trotter expansion (${{\rm{e}}}^{-i\pi {J}_{y}/2}\approx {({{\rm{e}}}^{-i\pi {J}_{{ya}}/2/2m}{{\rm{e}}}^{-i\pi {J}_{{yb}}/2/m}{{\rm{e}}}^{-i\pi {J}_{{ya}}/2/2m})}^{m}$ where $m$ is Trotter number) is necessary, with ${U}_{{ya}}$ having the form of ${{\rm{e}}}^{-i\pi {J}_{{ya}}/2/2m}$ and ${U}_{{yb}}$ having the form of ${{\rm{e}}}^{-i\pi {J}_{{yb}}/2/m}$ alternately evolving $m$ times, as shown in Fig. 2a.

As we see in the experimental results, Trotter expansion will induce a systematic error, which has been already considered in the program design, and does not affect the fidelity of the photonic processor.

### Calculation for the average Husimi distribution $Q\left(\theta ,\phi \right)$

The average is taken in both time and space. For a certain initial coherent state, we first derive the time average, by calculating the Husimi distribution of each ${\rho }_{i}$ after *i*th period of evolution (${R}_{i}\left(\theta ,\phi \right)$), and then their average ${R}_{{\rm{TA}}}\left(\theta ,\phi \right)=\frac{1}{1+n}\mathop{\sum }\nolimits_{i=0}^{n}{R}_{i}\left(\theta ,\phi \right)$. Next, we randomly choose $s$ = 35 initial coherent states in the whole phase space, and repeat the above-introduced evolutions and calculations, deriving $s$ different ${R}_{{\rm{TA}}}\left(\theta ,\phi \right)$. Now we need to take an average for these ${R}_{{\rm{TA}}}\left(\theta ,\phi \right)$, however, due to the symmetry of the space, this average will be all the same everywhere, which can not distinguish the regular region and chaotic region. Nevertheless, by observing the cases of $k=1$ and $k=12$, we still see an obvious difference between them in the distributions ${R}_{{\rm{TA}}}\left(\theta ,\phi \right)$. For the $k=1$ case, the profile of the island and sea in ${R}_{{\rm{TA}}}\left(\theta ,\phi \right)$ is approximately stable, but the ${R}_{{\rm{TA}}}$ values in island can sometimes larger than that in sea, and sometimes less than that. Then when an average is taken, both the ${R}_{{\rm{TA}}}$ values in island and sea will counteract, and erase out the profile. Whereas for the $k=12$ case, no stable profiles can be found. This difference well exhibit the chaotic property of this system when $k=12$, and in contrast, $k=1$ belongs to regular region. To show this chaotic behavior more clearly, we introduce an asymmetric transformation of ${R}_{{\rm{TA}}}\left(\theta ,\phi \right)$, i.e., ${{R\text{'}}}_{{\rm{TA}}}\left(\theta ,\phi \right)=(2{R}_{{\rm{TA}}}\left(\theta ,\phi \right)-1){\mathrm{sgn}}(2{R}_{{\rm{TA}}}\left({\theta }_{0},{\phi }_{0}\right)-1)$ with $\mathrm{sgn}(\cdot )$ being the sign function and $\left({\theta }_{0},{\phi }_{0}\right)$ representing a randomly chosen fixed point. For the regular case with stable profile, this transformation to a great extent stop the counteraction of ${{R\text{'}}}_{{\rm{TA}}}$ both in island and sea, and the profile is highlighted by taking the average of $s$ samples, i.e., $Q\left(\theta ,\phi \right)=\frac{1}{S}\mathop{\sum }\nolimits_{1}^{s}{{R\text{'}}}_{{\rm{TA}}}\left(\theta ,\phi \right)$. Whereas for the chaotic case which does not have a stable profile, the same process still can not stop the counteraction and the average $Q\left(\theta ,\phi \right)$ is none the less always the same everywhere. We note that the choice of $\left({\theta }_{0},{\phi }_{0}\right)$ basically does not affect the main conclusion. Additional information can be found in Supplementary Information.

### Fidelity decay as the indicator of quantum signature of chaos and quantitative features in Fermi golden region

The classical chaos can be heuristically determined by the effect of the tiny perturbation which will dramatically change the evolution results. Along the similar idea, the effect of the perturbation can be measured by the fidelity between the evolved states with ($\left|{\phi }_{n} \rangle ={U}_{P}^{n}\right|{\phi }_{0} \rangle$) and without ($|{\phi }_{n}^{{\prime} } \rangle ={U}^{n}|{\phi }_{0} \rangle$) the perturbation ($P$) for the given initial state $|{\phi }_{0} \rangle$, i.e.,

3 $${F}_{n}={{\rm{|}}\left\langle {\phi }_{n},|,{\phi }_{n}^{{\prime} }\right\rangle {\rm{|}}}^{2}$$

The effect of the initial state can be removed by taking average on the initial state in the whole space. According to RMT in quantum chaos^61–63^, the average fidelity should exhibit exponential-decay behavior, i.e.,

4 $${\bar{F}}_{n}\simeq \exp (-\varGamma n)$$

where $\varGamma$ represents the decay rate with the form of $\varGamma =\xi (j){\delta }^{2}$, and $\xi (j)$ is related to the angular momentum quantum number $j$. $\delta$ is the perturbation strength in the perturbation Hamiltonian $P={{\rm{e}}}^{-i\delta {J}_{z}}$, and $\delta$ then can also be understood as the phase shifts of each eigenstate of ${J}_{z}$^66^. Under this situation, $\xi (j)$ only depends on the eigenvalues of ${J}_{z}$ (i.e., only $j$-dependent), thus the decay rate of average fidelity will be proportional to ${\delta }^{2}$. Since the detailed behavior of the decay rate is derived from RMT, the observation of the corresponding behavior not only can be used to distinguish the regular and chaos region of the system, but also can be used to support RMT behind quantum chaos.

Generally, there are different chaos regions in the chaotic system (may suggest different theories behind them), such as, Fermi gold region and Lyapunov region^75^ which can be distinguished by the dependence of the decay rate on the kick strength $k$. In the Fermi golden region, the decay rate is independent of the parameter $k$, however, in the Lyapunov region the decay rate will change with $k$.

In our programmable processor, we observed the exponential decay of the average fidelity and its decay rate is scaled with the perturbation strength $\delta$ as ${\delta }^{2}$ with $k=12$. These results definitely confirmed that the system is in chaos region and strongly support that RMT is valid in chaotic system. Our further observation that the decay rate keeps all the same for $k=12,14.3,16.6$, suggests that the QKM system with $k\in [12,16.6]$ is in Fermi golden region.

### Supplementary information

Supplementary Information for A von-Neumann-like photonic processor and its application in studying quantum signature of chaos
