# Supplementary material for: A von-Neumann-like photonic processor and its application in studying quantum signature of chaos
Source: Light Sci Appl. 2024 Mar 14;13:74. doi: 10.1038/s41377-024-01413-5 (PMC10940704; doi:10.1038/s41377-024-01413-5)
Supplement: Supplementary file 1 — Supplementary Information for A von-Neumann-like photonic processor and its application in studying quantum signature of chaos [file 41377_2024_1413_MOESM1_ESM.pdf]

Supplementary Information for
A von-Neumann-like photonic processor and its application in studying quantum signature of chaos

Shang Yu,[1,2,3,4,5] Wei Liu,[1,2,3] Si-Jing Tao,[1,2,3] Zhi-Peng Li,[1,2,3] Yi-Tao Wang,[1,2,3] Zhi-Peng Zhong,[4] Raj B. Patel,[5,6] Yu Meng,[1,2,3] Yuan-Ze Yang,[1,2,3] Zhao-An Wang,[1,2,3] Nai-Jie Guo,[1,2,3] Xiao-Dong Zeng,[1,2,3] Zhe Chen,[1,2,3] Liang Xu,[4] Ning Zhang,[4] Xiao Liu,[1,2,3] Mu Yang,[1,2,3] Wen-Hao Zhang,[1,2,3] Zong-Quan Zhou,[1,2,3] Jin-Shi Xu,[1,2,3] Jian-Shun Tang,[1,2,3,*] Yong-Jian Han,[1,2,3,†] Chuan-Feng Li,[1,2,3,‡] and Guang-Can Guo[1,2,3]

[1]*CAS Key Laboratory of Quantum Information, University of Science and Technology of China, Hefei 230026, China.*
[2]*CAS Center for Excellence in Quantum Information and Quantum Physics, University of Science and Technology of China, Hefei 230026, China.*
[3]*Hefei National Laboratory, University of Science and Technology of China, Hefei 230088, China.*
[4]*Research Center for Quantum Sensing, Zhejiang Lab, Hangzhou 310000, China.*
[5]*Quantum Optics and Laser Science, Blackett Laboratory, Imperial College London, Prince Consort Rd, London SW7 2AZ, United Kingdom.*
[6]*Clarendon Laboratory, Department of Physics, Oxford University, Parks Road OX1 3PU Oxford, United Kingdom.*

I. KICKED-TOP MODEL AND ITS DECOMPOSITION TO QUANTUM-GATE SERIES FOR PROGRAMMING.

In the main text, we have introduced the quantum kicked-top model, which is governed by a periodic Hamiltonian expressed as Eq. (1) (in main text). J contained in this Hamiltonian is the angular momentum ($J^2 = j(j+1)$). We choose $j = 6$, then the angular momentum operators J_y and J_z can be explicitly expressed as

$$J_y = \begin{pmatrix}
0 & -i\sqrt{3} & 0 & 0 & 0 & 0 & 0 & 0 & 0 & 0 & 0 & 0 & 0 \\
i\sqrt{3} & 0 & -i\sqrt{\frac{11}{2}} & 0 & 0 & 0 & 0 & 0 & 0 & 0 & 0 & 0 & 0 \\
0 & i\sqrt{\frac{11}{2}} & 0 & -i\sqrt{\frac{15}{2}} & 0 & 0 & 0 & 0 & 0 & 0 & 0 & 0 & 0 \\
0 & 0 & i\sqrt{\frac{15}{2}} & 0 & -i3 & 0 & 0 & 0 & 0 & 0 & 0 & 0 & 0 \\
0 & 0 & 0 & i3 & 0 & -i\sqrt{10} & 0 & 0 & 0 & 0 & 0 & 0 & 0 \\
0 & 0 & 0 & 0 & i\sqrt{10} & 0 & -i\sqrt{\frac{21}{2}} & 0 & 0 & 0 & 0 & 0 & 0 \\
0 & 0 & 0 & 0 & 0 & i\sqrt{\frac{21}{2}} & 0 & -i\sqrt{\frac{21}{2}} & 0 & 0 & 0 & 0 & 0 \\
0 & 0 & 0 & 0 & 0 & 0 & i\sqrt{\frac{21}{2}} & 0 & -i\sqrt{10} & 0 & 0 & 0 & 0 \\
0 & 0 & 0 & 0 & 0 & 0 & 0 & i\sqrt{10} & 0 & -i3 & 0 & 0 & 0 \\
0 & 0 & 0 & 0 & 0 & 0 & 0 & 0 & i3 & 0 & -i\sqrt{\frac{15}{2}} & 0 & 0 \\
0 & 0 & 0 & 0 & 0 & 0 & 0 & 0 & 0 & i\sqrt{\frac{15}{2}} & 0 & -i\sqrt{\frac{11}{2}} & 0 \\
0 & 0 & 0 & 0 & 0 & 0 & 0 & 0 & 0 & 0 & i\sqrt{\frac{11}{2}} & 0 & -i\sqrt{3} \\
0 & 0 & 0 & 0 & 0 & 0 & 0 & 0 & 0 & 0 & 0 & i\sqrt{3} & 0
\end{pmatrix} \quad (1)$$

*Electronic address: tjs@ustc.edu.cn
†Electronic address: smhan@ustc.edu.cn
‡Electronic address: cfli@ustc.edu.cn

and

$$J_z = \begin{pmatrix} 6 & 0 & 0 & 0 & 0 & 0 & 0 & 0 & 0 & 0 & 0 & 0 & 0 \\ 0 & 5 & 0 & 0 & 0 & 0 & 0 & 0 & 0 & 0 & 0 & 0 & 0 \\ 0 & 0 & 4 & 0 & 0 & 0 & 0 & 0 & 0 & 0 & 0 & 0 & 0 \\ 0 & 0 & 0 & 3 & 0 & 0 & 0 & 0 & 0 & 0 & 0 & 0 & 0 \\ 0 & 0 & 0 & 0 & 2 & 0 & 0 & 0 & 0 & 0 & 0 & 0 & 0 \\ 0 & 0 & 0 & 0 & 0 & 1 & 0 & 0 & 0 & 0 & 0 & 0 & 0 \\ 0 & 0 & 0 & 0 & 0 & 0 & 0 & 0 & 0 & 0 & 0 & 0 & 0 \\ 0 & 0 & 0 & 0 & 0 & 0 & 0 & -1 & 0 & 0 & 0 & 0 & 0 \\ 0 & 0 & 0 & 0 & 0 & 0 & 0 & 0 & -2 & 0 & 0 & 0 & 0 \\ 0 & 0 & 0 & 0 & 0 & 0 & 0 & 0 & 0 & -3 & 0 & 0 & 0 \\ 0 & 0 & 0 & 0 & 0 & 0 & 0 & 0 & 0 & 0 & -4 & 0 & 0 \\ 0 & 0 & 0 & 0 & 0 & 0 & 0 & 0 & 0 & 0 & 0 & -5 & 0 \\ 0 & 0 & 0 & 0 & 0 & 0 & 0 & 0 & 0 & 0 & 0 & 0 & -6 \end{pmatrix} \tag{2}$$

This system has an equivalent Floquet representation written as Eq. (2) (in main text), which includes two subevolutions: $e^{-i\pi J_y/2}$ and $e^{-ikJ_z^2/j}$.

The subevolution $e^{-ikJ_z^2/j}$ is simply assigned to a phase gate. The other subevolution $e^{-i\pi J_y/2}$ is handled as follows. $J_y = J_{ya} + J_{yb}$, with

$$J_{ya} = \begin{pmatrix} 0 & -i\sqrt{3} & 0 & 0 & 0 & 0 & 0 & 0 & 0 & 0 & 0 & 0 & 0 \\ i\sqrt{3} & 0 & 0 & 0 & 0 & 0 & 0 & 0 & 0 & 0 & 0 & 0 & 0 \\ 0 & 0 & 0 & -i\sqrt{\frac{15}{2}} & 0 & 0 & 0 & 0 & 0 & 0 & 0 & 0 & 0 \\ 0 & 0 & i\sqrt{\frac{15}{2}} & 0 & 0 & 0 & 0 & 0 & 0 & 0 & 0 & 0 & 0 \\ 0 & 0 & 0 & 0 & 0 & -i\sqrt{10} & 0 & 0 & 0 & 0 & 0 & 0 & 0 \\ 0 & 0 & 0 & 0 & i\sqrt{10} & 0 & 0 & 0 & 0 & 0 & 0 & 0 & 0 \\ 0 & 0 & 0 & 0 & 0 & 0 & 0 & -i\sqrt{\frac{21}{2}} & 0 & 0 & 0 & 0 & 0 \\ 0 & 0 & 0 & 0 & 0 & 0 & i\sqrt{\frac{21}{2}} & 0 & 0 & 0 & 0 & 0 & 0 \\ 0 & 0 & 0 & 0 & 0 & 0 & 0 & 0 & 0 & -i3 & 0 & 0 & 0 \\ 0 & 0 & 0 & 0 & 0 & 0 & 0 & 0 & i3 & 0 & 0 & 0 & 0 \\ 0 & 0 & 0 & 0 & 0 & 0 & 0 & 0 & 0 & 0 & 0 & -i\sqrt{\frac{11}{2}} & 0 \\ 0 & 0 & 0 & 0 & 0 & 0 & 0 & 0 & 0 & 0 & i\sqrt{\frac{11}{2}} & 0 & 0 \\ 0 & 0 & 0 & 0 & 0 & 0 & 0 & 0 & 0 & 0 & 0 & 0 & 0 \end{pmatrix} \tag{3}$$

and

$$J_{yb} = \begin{pmatrix} 0 & 0 & 0 & 0 & 0 & 0 & 0 & 0 & 0 & 0 & 0 & 0 & 0 \\ 0 & 0 & -i\sqrt{\frac{11}{2}} & 0 & 0 & 0 & 0 & 0 & 0 & 0 & 0 & 0 & 0 \\ 0 & i\sqrt{\frac{11}{2}} & 0 & 0 & 0 & 0 & 0 & 0 & 0 & 0 & 0 & 0 & 0 \\ 0 & 0 & 0 & 0 & -i3 & 0 & 0 & 0 & 0 & 0 & 0 & 0 & 0 \\ 0 & 0 & 0 & i3 & 0 & 0 & 0 & 0 & 0 & 0 & 0 & 0 & 0 \\ 0 & 0 & 0 & 0 & 0 & 0 & -i\sqrt{\frac{21}{2}} & 0 & 0 & 0 & 0 & 0 & 0 \\ 0 & 0 & 0 & 0 & 0 & i\sqrt{\frac{21}{2}} & 0 & 0 & 0 & 0 & 0 & 0 & 0 \\ 0 & 0 & 0 & 0 & 0 & 0 & 0 & 0 & -i\sqrt{10} & 0 & 0 & 0 & 0 \\ 0 & 0 & 0 & 0 & 0 & 0 & 0 & i\sqrt{10} & 0 & 0 & 0 & 0 & 0 \\ 0 & 0 & 0 & 0 & 0 & 0 & 0 & 0 & 0 & 0 & -i\sqrt{\frac{15}{2}} & 0 & 0 \\ 0 & 0 & 0 & 0 & 0 & 0 & 0 & 0 & 0 & i\sqrt{\frac{15}{2}} & 0 & 0 & 0 \\ 0 & 0 & 0 & 0 & 0 & 0 & 0 & 0 & 0 & 0 & 0 & 0 & -i\sqrt{3} \\ 0 & 0 & 0 & 0 & 0 & 0 & 0 & 0 & 0 & 0 & 0 & i\sqrt{3} & 0 \end{pmatrix} \tag{4}$$

$e^{-i\pi J_{ya}/2}$ and $e^{-i\pi J_{yb}/2}$ can be further decomposed into a series of two-level gates between the adjacent modes. However, since J_{ya} and J_{yb} is not commuting, Trotter expansion ($e^{-i\pi J_y/2} \approx (e^{-i\pi J_{ya}/2/2m} e^{-i\pi J_{yb}/2/m} e^{-i\pi J_{ya}/2/2m})^m$

where m is Trotter number) is needed for the reconstruction of $e^{-i\pi J_y/2}$, with $U_{ya} = e^{-i\pi J_{ya}/2/2m}$ and $U_{yb} = e^{-i\pi J_{yb}/2/m}$ alternately evolving m times, as shown in Fig. 2a in main text. By programming these quantum gates in sequence, we can simulate the kicked-top evolution in each period.

II. REALIZATION OF ARBITRARY TWO-MODE UNITARY GATE USING 3 EOMS.

EOM2-4 (electro-optic modulator) in Fig. 1 of main text can be driven fast and accurately to provide 3 arbitrary phases ϕ_1, ϕ_2 and ϕ_3 with range of $[-\pi, \pi]$ (full-λ). Then these three EOMs assisted with several half-wave plates (HWPs) can construct a two-mode gate

$$U_2 = U_\varphi U_{\phi_3} U_{\text{HWP@22.5}^\circ} U_{\phi_2} U_{\text{HWP@22.5}^\circ} U_{\phi_1}$$

where $U_\varphi = \begin{pmatrix} e^{i\varphi} & 0 \\ 0 & e^{i\varphi} \end{pmatrix}$ denotes the global phase, $U_{\phi_j} = \begin{pmatrix} e^{i\phi_j} & 0 \\ 0 & 1 \end{pmatrix}$ $(j = 1 \sim 3)$ are the fast phase gates realized by EOM2-4, and $U_{\text{HWP@22.5}^\circ} = -\frac{1}{\sqrt{2}} \begin{pmatrix} 1 & 1 \\ 1 & -1 \end{pmatrix}$. U_2 can simulate arbitrary two-mode gates. To illustrate this, we take an example of the first two-mode subevolution of $U_{ya} = e^{-i\pi J_{ya}/2/2m}$ after Trotter expansion with $m = 3$. It is $U_{ya12} = \exp\left[-i\frac{\pi}{12}\begin{pmatrix} 0 & -i\sqrt{3} \\ i\sqrt{3} & 0 \end{pmatrix}\right]$. When $\varphi = -0.45345$, $\phi_1 = -1.5708$, $\phi_2 = 0.9069$, $\phi_3 = 1.5708$, U_{ya12} can be well simulated by U_2 (In practice, the target matrix may contains a space transformation due to the basis redefinition, e.g., the polarization exchange). The global phase φ can be compensated in U_{ϕ_1} of the next loop. Note that, as this circuit is isomorphic to photonic chips, using the construction methods there, arbitrary gates with more modes can be constructed directly without Trotter expansion, but the latter will help cost less resources and has a clear physical picture.

III. FIDELITY OF ONE PERIOD.

As described in main text, our photonic processor has an enough high fidelity. Here we take the first period of evolution in Fig. 2a and a simple input state of $\rho_i = |3\rangle\langle 3|$ ($|3\rangle$ represents the third mode among 13 modes) as an example. ρ_i goes through this single period of evolution, and we measure and reconstruct the evolved state after the evolution, i.e., ρ_f. Then we calculate the fidelity between this experimental result and the ideally-evolved state $\rho_{f,th}$ using $F = \text{Tr}\sqrt{\rho_{f,th}^{1/2}\rho_f\rho_{f,th}^{1/2}}$. The matrices of $\rho_{f,th}$ and ρ_f are shown in Fig. S1, and the fidelity between them is calculated to be 0.992.

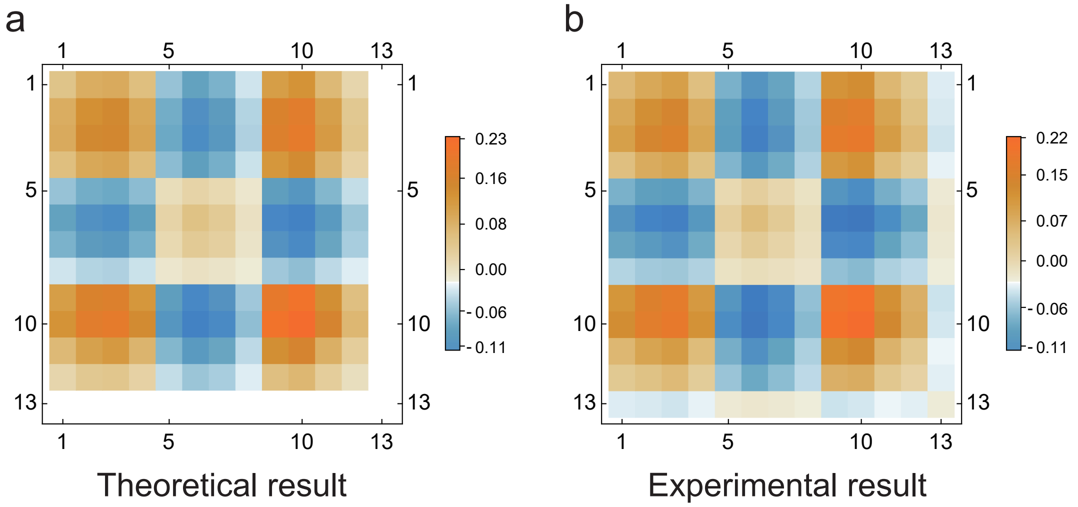

FIG. S1: The matrices of evolved states after the first-period evolution. (a) Matrix of the theoretical evolved state (errors of Trotter expansion are included here). (b) Matrix of the experimental evolved state.

IV. PHOTONS FOR THE CHAOS EXPERIMENT.

As discussed in main text, our work focuses on the programmable circuit primarily, and the photon sources can be various like single or entangled photons, squeezed state or cluster state, etc. For the experiment of studying quantum signature of chaos, a 825-nm light is chopped by a home-made chopper to obtain a pulse with width of 20 ns and high signal-to-noise ratio. The chopper contains 3 double-passed acousto-optic modulators (AOMs), and its repetition frequency is 10 kHz. Every pass will obtain an extinction ratio of approximate 10^3, thus the total extinction ratio will reach approximately 10^{18}.

V. PHOTON LOSSES.

The single-loop photon transmission efficiency is approximately 0.494, which can be derived by multiplying the efficiency of each individual element in the round trip. They consist of the efficiencies of the fibers (including the collection efficiencies from free space to fibers and the transmission efficiencies in the fibers): two 20-m fiber, 0.919 and 0.926, and the long fiber, 0.789; the transmission efficiencies of EOM1 (0.971), EOM2 (0.975), EOM3 (0.967), EOM4 (0.973), EOM5 (0.969), and of all other optical elements including an AOM (approximately 0.930). Therefore, the single-loop transmission efficiency can be calculated as $0.494 \approx (0.975^2 \times 0.967^2 \times 0.973^2) \times (0.971 \times 0.969) \times [0.789 \times 0.919 \times 0.926)] \times 0.930$.

The maximal number of evolution periods in our experiment is 6, which corresponds to 49 loops (after appropriate combination of some loops, which will be discussed later). Moreover, when the signal is send out of the loops by the AOM, the diffraction efficiency is 0.724. The total efficiency is approximately $0.494^{49} \times 0.724 \approx 7.12 \times 10^{-16}$.

The extinction ratio of 3 AOMs is approximately 10^{18}, which is enough for this evolution, and still can achieve around 10^2 noise and signal ratio.

The main loss comes from the delay line. On one hand, we can change the wavelength to communication band, which processes a much smaller loss; on the other hand, we can use a other memories, for example, the one reported by Hsiao et al. [1], which has an efficiency of $\sim 90\%$ at storage time of $\sim 10\ \mu$s.

VI. THE FIRST PROGRAM: HUSIMI DISTRIBUTION.

As introduced in main text, the phase space is represented by Husimi distribution. A coherent state with angular momentum j can be written as [2, 3]

$$|\theta,\phi\rangle = \sum_{m=-j}^{j} \binom{2j}{m+j}^{\frac{1}{2}} (\sin\frac{\theta}{2})^{j+m} \times (\cos\frac{\theta}{2})^{j-m} e^{-i(j+m)\phi} |jm\rangle \tag{5}$$

then the Husimi distribution can be obtained by calculating the expectation value of the density operator on the coherent-state bases

$$R(\theta,\phi) = \langle\theta,\phi|\rho|\theta,\phi\rangle \tag{6}$$

VII. THE FIRST PROGRAM: SUPPLEMENTARY DATA.

The first program is designed to observe the regular and chaotic behaviors in phase space, which will give the visual pictures of the system states and clearly show the difference between regularity and quantum signature of chaos. In this program, 13 temporal modes are used to carry out the U evolution, and after several periods ($n = 4$ here), the system will show or not show chaotic phenomenon depending on different programming parameters, for example, when $k = 12$, the quantum behavior of chaos is supposed to show, and when $k = 1$, the system state is regular.

As described in main text, the chaotic behavior is an average effect of many evolutions both in time and space. We first derive the time average by detecting the Husimi distribution after each evolution period (with index i) and calculating their average $R_{TA}(\theta,\phi) = \frac{1}{1+n}\sum_{i=0}^{n} R_i(\theta,\phi)$. Then to derive the average in space, we should randomly choose $s(= 35$ here) initial coherent states in the whole phase space, and repeat the above evolutions and time-averaged calculations, deriving s different $R_{TA}(\theta,\phi)$. Fig. S2 shows 3 typical results of $R_{TA}(\theta,\phi)$ for both $k = 1$ and $k = 12$ cases, from the viewangle of $+y$. From these results, we can clearly find that, in the $k = 1$ case, the profile of the island and sea in $R_{TA}(\theta,\phi)$ is approximately stable, but the R_{TA} values in island can sometimes larger than

that in sea, and sometimes less than that. If we take an average directly, both the R_{TA} values in island and sea will counteract, and erase out the profile. Whereas in the $k = 12$ case, no stable profiles can be found, and if a direct average is taken, profile is either not seen. This difference well exhibits the chaotic property of this system when $k = 12$, and in contrast, the regularity when $k = 1$, but the direct average in space can not distinguish them.

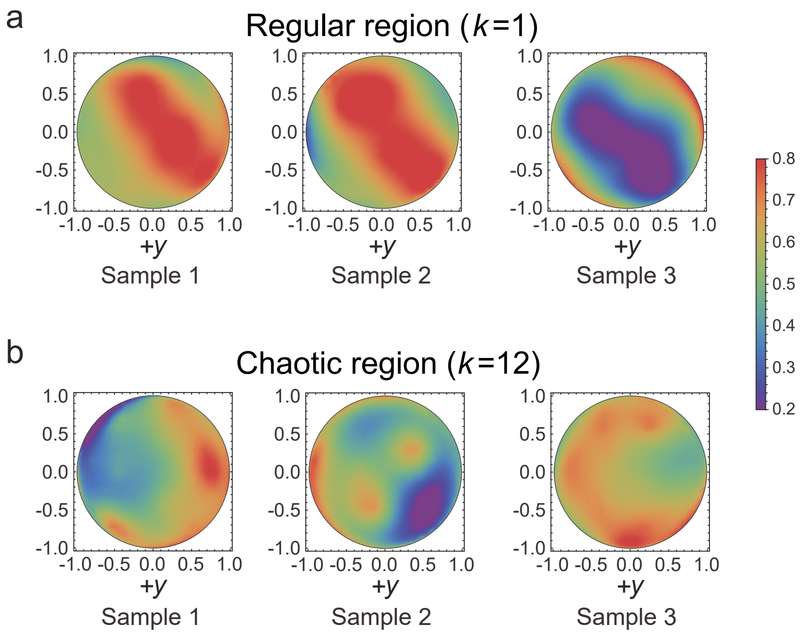

FIG. S2: Experimental results for time-averaged Husimi distribution ($R_{TA}(\theta, \phi)$) of three typical samples.

Therefore, we introduce an asymmetric transformation of $R_{TA}(\theta, \phi)$, i.e., $R'_{TA}(\theta, \phi) = (2R_{TA}(\theta, \phi) - 1)\mathrm{sgn}(2R_{TA}(\theta_0, \phi_0) - 1)$ with $\mathrm{sgn}(\cdot)$ being the sign function and (θ_0, ϕ_0) representing a randomly chosen fixed point. The choice of (θ_0, ϕ_0) basically does not affect the main conclusion. For the regular case with stable profile, this transformation to a great extent stop the counteraction of R'_{TA} both in island and sea, and the profile is highlighted by taking the average of s samples, i.e., $Q(\theta, \phi) = \frac{1}{s}\sum_1^s R'_{TA}$. Whereas for the chaotic case which does not have a stable profile, the same process still can not stop the counteraction and the average $Q(\theta, \phi)$ none the less has no obvious profile. The experimental and ideal (calculated using $s = 10000$ samples) results are shown in Fig. S3, and Figs. 2b,c in main text are included in this figure. By comparing the $+y$ results in both Fig. S2 and S3, we can clearly see the role of this asymmetric transformation. Table S1 is a diagram of the calculation process of $Q(\theta, \phi)$.

VIII. THE SECOND PROGRAM: SUPPLEMENTARY DETAILS ON THE ANCILLA STATE PREPARATION AND DATA READOUT.

As shown in Fig. 3a in main text, an ancilla qubit encoded on polarization mode is operated by R_x gate and then transformed to time mode, before it is sent into the processor. This is the state preparation of ancilla for the program, and the details are shown in Fig. S4a. The first polarizing beam splitter (PBS) initializes the horizontal-polarization state $|H\rangle$, then R_x rotates this state. After that, two PBS's and a 10-m fiber separate the horizontal- and vertical-polarization modes by 50 ns, and the following 22.5°-HWP and PBS eliminate the polarization mode and leaving the state encoded on time mode. This process will cost half of the photons.

Similarly, after the time-mode-encoded state (which contains the ancilla) is output from the processor, the ancilla is converted back to polarization mode and goes through another R_x gate before an expectation-value measurement on σ_z. This is the data-readout process and detailedly shown in Fig. S4b. The HWP at 22.5° and the following two PBS's with 10-m fiber recombine the time mode to recover the polarization qubit with one half probability, then the polarization qubit is R_x-operated and σ_z-detected. Note that these two 10-m fibers are also double-core fiber which is phase-stable.

Table S1: Calculation of the average of the transformed Husimi distribution $Q(\theta, \phi)$.

Settings: unitary evolution $U = e^{-i\pi J_y/2} e^{-ikJ_z^2/j}$, angular momentum operators J_y, J_z, angular momentum quantum number j, kicking strength k, number of evolution periods n, a randomly selected fixed point (θ_0, ϕ_0)

Input: initial coherent state $|\theta, \phi\rangle$, number of initial states s

for $p \in 1 \rightarrow s$ **do**
  randomly initialize the initial state
  $\theta_{\rm ini}[p]$ = RandomReal[0, π]
  $\phi_{\rm ini}[p]$ = RandomReal[-π, π]
  for $q \in 0 \rightarrow n$ **do**
    perform evolution $U^q |\theta_{\rm ini}[p], \phi_{\rm ini}[p]\rangle$ and calculating $R[p][q](\theta, \phi)$
  end for
  calculate time average $R_{TA}[p](\theta, \phi) = \frac{1}{n+1} \sum_{q=0}^{n} R[p][q](\theta, \phi)$
  if $2R_{TA}(\theta_0, \phi_0) - 1 \geq 0$ **then**
    $R'_{TA}[p](\theta, \phi) = 2R_{TA}[p](\theta, \phi) - 1$
  else
    $R'_{TA}[p](\theta, \phi) = -(2R_{TA}[p](\theta, \phi) - 1)$
  end if
end for
calculate the final average $Q(\theta, \phi) = \frac{1}{s} \sum_{p=1}^{s} R'_{TA}[p](\theta, \phi)$

Output: $Q(\theta, \phi)$

IX. THE SECOND PROGRAM: PULSE DIAGRAM.

To illustrate our quantum circuit clearly, we present a sketched pulse diagram during the whole second program in Fig. S5. This diagram will be introduced as three parts: the state preparation, the evolution circuit and the data readout, as compared with Fig. 3a and Fig. 2a in main text.

For the state preparation, 13 temporal modes with H state (horizontal polarization) are initialized. The time separation of adjacent time modes is 100 ns and the effective pulse width is 20 ns as mentioned previously. Then the photons go through a R_x gate on the polarization mode which acts as the ancilla qubit. A 50-ns time delay is performed on V (vertical) polarization, and totally 26 temporal modes are created. These time modes contain all the amplitude and phase information, but with a polarization label. To eliminate this label, we use a 22.5°-HWP and a PBS to finish the state preparation with all modes occupying H polarization. Then this 26-mode state is sent into the quantum circuit.

The circuit is constituted of individual periods. In this program, $n(=5)$ or 6 periods are carried out. Each period is further recognized as three subevolutions: controlled-$P = e^{-i\delta J_z}$, $e^{-ikJ_z^2/j}$ and $e^{-i\pi J_y/2}$, as discussed previously and in main text. For controlled-$e^{-i\delta J_z}$ subevolution which is represented by the blue box, EOM1 keeps the polarization as H state for the first period and transforms V to H in the following periods. Then the pulses with even index are applied with a $e^{-i\delta J_z}$ phase gate by EOM2-4 (indicated using red solid triangles), and the odd-index pulses are not. EOM5 transform all the pulses to V polarization before they are sent into the next loop. For $e^{-ikJ_z^2/j}$ subevolution represented by green box, it is also a phase gate, but applied to all the time pulses.

For $e^{-i\pi J_y/2}$ subevolution, it can be decomposed by Trotter expansion as discussed previously, i.e., $e^{-i\pi J_y/2} \approx (U_{ya}U_{yb}U_{ya})^m = (e^{-i\pi J_{ya}/2/2m} e^{-i\pi J_{yb}/2/m} e^{-i\pi J_{ya}/2/2m})^m$. Here, m is set to 3, therefore, this subevolution can be further decomposed into 3 subperiod with each represented by a gray box. We only draw the first subperiod which contains three subsubevolutions $(U_{ya}U_{yb}U_{ya})$ in the first gray box, and the other two gray boxes are the repeats of the first one. According to the state preparation process, these 26 pulses are naturally group into 13 pairs. For the first U_{ya}, EOM1 transforms the pulse pairs with even index to H polarization, and a V-delay combine the adjacent pairs of pulses, then EOM2-4 perform U_{ya} on these combined modes (indicated in figure using red solid triangles). After that, an H-delay realized by adding two HWPs to the V-delay setup (see Fig. 1 in main text) separates the H and V pulses again, and EOM5 transforms all the H state to V before sending them into the next loop. For U_{yb}, it is similar to U_{ya}, except that it is the odd-index pairs who are transformed to H by EOM1; and for the second U_{ya}, it is the same as the first one.

After the whole evolution, the state encoded on 26 V-polarized time modes are sent out of the circuit for data readout. A 22.5°-HWP and a V-delay recombine the odd- and even-index pulses and transfer the information back to polarization mode as the ancilla qubit. These recombined modes are then post-selected and go through another R_x gate. Finally, the result (average fidelity) is directly readout by measuring the expectation value of σ_z on the ancilla.

In this analysis for the chaos-related program, we have not considered the Hong-Ou-Mandel effect since it is not necessary for this quantum task, but we want to note that this effect is also allowable when other programs are carried

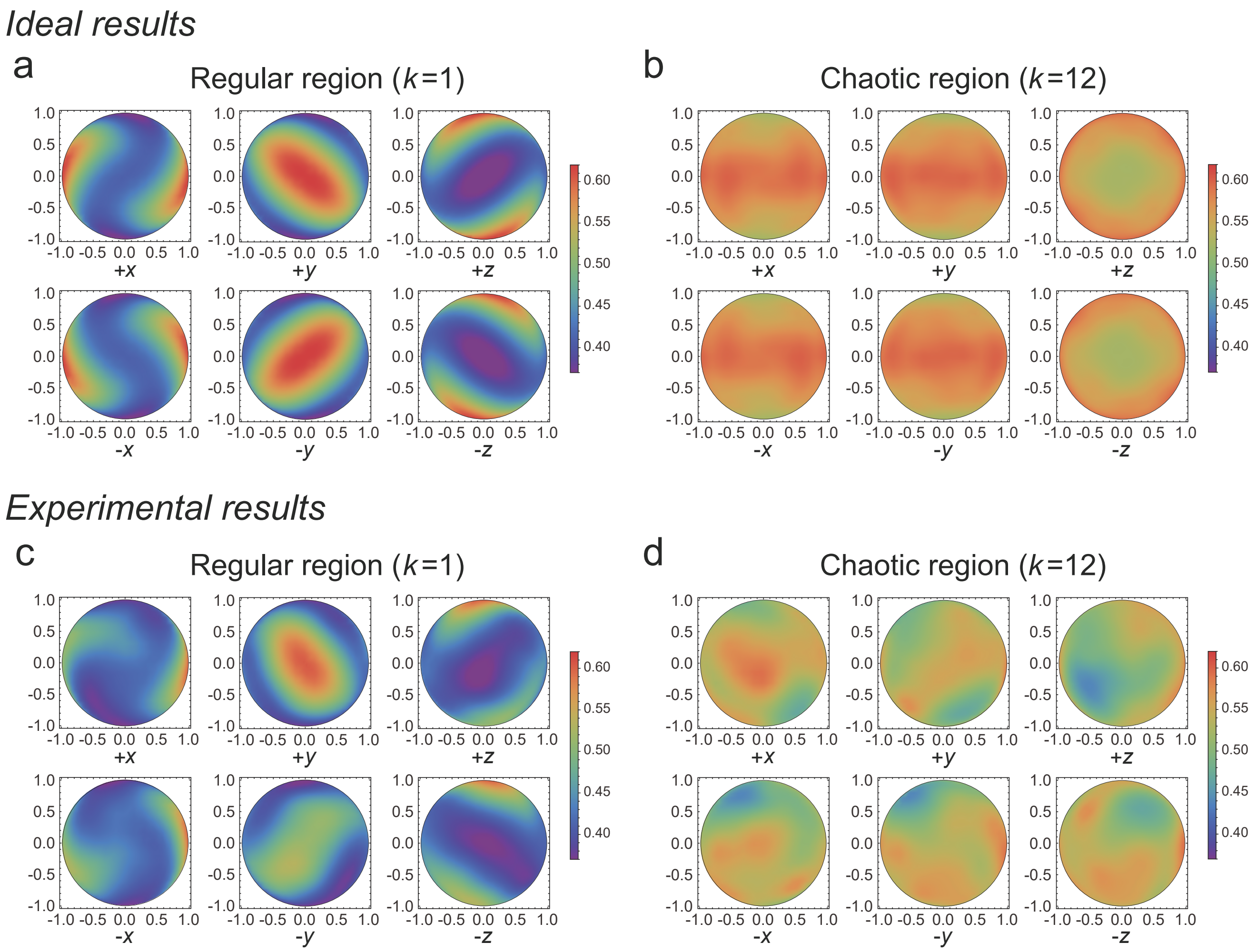

FIG. S3: Ideal and experimental results of $Q(\theta,\phi)$ under regular evolution ($k=1$) and chaotic evolution ($k=12$).

out by using the input photon source and state possessing identical photons if necessary.

X. THE SECOND PROGRAM: CONTROL SEQUENCE FOR ONE PERIOD.

Fig. S6a depicts the logic diagram of this program for $n=1$ period on the left. It contains three subevolutions: controlled-$e^{-i\delta J_z}$, $e^{-ikJ_z^2/j}$ and $e^{-i\pi J_y/2}$, as discussed previously, and the symbols are the same as that in Fig. 2a of main text. We find that the subevolutions controlled-$e^{-i\delta J_z}$ and $e^{-ikJ_z^2/j}$ are both phase gates and can be combined, the subsubevolutions U_{ya} from the adjacent subperiods can also be combined. The combined diagram is shown on the right. For one period, 8 loops are carried out, and in our experiment, at most 6 periods are performed, which corresponds to 48 loops. As we have mentioned previously, the simulation of two-level gate using 3 EOMs will induce a phase shift, and this phase can be compensated in the next loop, but for the last loop (namely, the 48th loop), we need another loop to compensate its phase shift, therefore, the total loop number is 49. The last phase-compensation loop is not drawn in Figs. S5 and S6.

Fig. S6b is a sketch of the control sequence for one period based on the combined logic diagram. The expressions of the corresponding subevolutions and their positions in the combined diagram are shown on the right. It will be better understood by referring to Fig. S5. Due to the input polarization states of the first and following periods are opposite, the control signals for the first line of EOM1 are also opposite. The solid line corresponds to the first period, and the dashed line corresponds to others. This control sequence is compiled according to the quantum task and stored in the instruction memory. We note that it is only guide for eyes, and has not been used in the practical experiment.

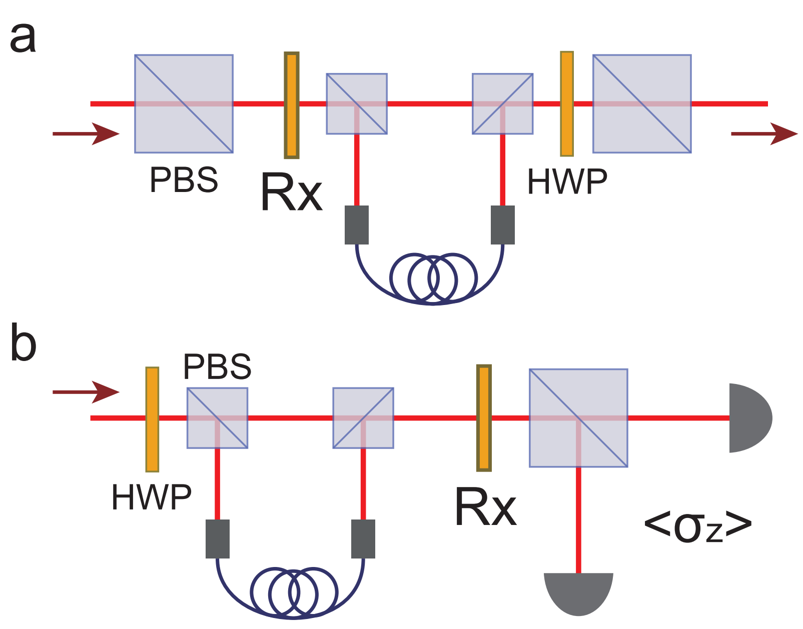

FIG. S4: (a) State preparation of ancilla and (b) data readout for the second program. The HWPs are set at 22.5°.

XI. THE SECOND PROGRAM: SUPPLEMENTARY DATA FOR FIDELITY DECAY WITH VARIOUS δ.

Fig. S7 is the supplementary data for Fig. 3c in main text. The perturbation strength δ is varying when $k = 12$, and $\mathcal{F}$ is plotted with the period index. These data are then linearly fitted, with the slope denoted as $c(j)\Delta$ ($c(j)$ is a constant). The dashed lines and the corresponding shadow areas represent the linear fits and corresponding fitting errors, which constitute the data points (hollow circles) and error bars in Fig. 3c.

XII. PHASE STABILIZATION BY USING DOUBLE-CORE OPTICAL FIBER.

As shown in Fig. 1b of main text, we use two optical fibers to combine adjacent modes and then re-separate them after the arbitrary two-level gate realized by 3 EOMs. To ensure the phase stability, these two fibers are actually encapsulated as an individual double-core fiber. This structure makes the two fiber cores go through the same environment, and keep the phase stability without additional phase-locking circuit.

In order to quantitatively analyse the phase stability, we observe the number of photons measured on the $|H\rangle + |V\rangle$ basis after interference between adjacent modes during a period of time. The stability of the phase can be quantitatively analysed by examining the fluctuation amplitude of photon numbers.

Fig. S8 illustrates the photon count fluctuation on the $|H\rangle + |V\rangle$ basis over approximately 10 minutes. It is evident that there is negligibly significant fluctuation in photon counting during these 10 minutes, indicating that the phase stability is quite reliable. This stability is crucial for the smooth conduct of our experiment.

Additionally, we will explain why we opted not to use the more stable free-space delay line mentioned in Ref. [4] for this experiment, and instead chose to employ a double-core fiber.

There are two primary reasons for using a 7.5-meter free space delay line in our upgraded version, as detailed in Ref. [4]. The first reason pertains to the quality of interference between two time-bins. In Gaussian Boson Sampling (GBS) experiments, the pulse duration is approximately 2 picoseconds, with a 25-nanosecond time difference between two time-bins. Therefore, we utilize a $\sim$7.5-meter free space delay line. A fibre delay line is not suitable in this context due to the relatively large dispersion effect compared to the 2-picosecond pulse duration. In current experiment, each pulse, chopped from a CW laser, has a duration of 20 nanoseconds and a 100-nanosecond pitch between each neighbouring time-bin. This necessitates a $\sim$30-meter free space delay line, which is challenging to implement without using a Herriot cell. By contrast, a fibre delay line is much more convenient for this purpose, and the larger pulse duration ensures high interference fidelity using a 20-meter fibre.

The second consideration is loss. In GBS experiments, photon loss significantly impacts the quantum characteristics of the experiments, potentially preventing the demonstration of computational advantages. A free space delay line

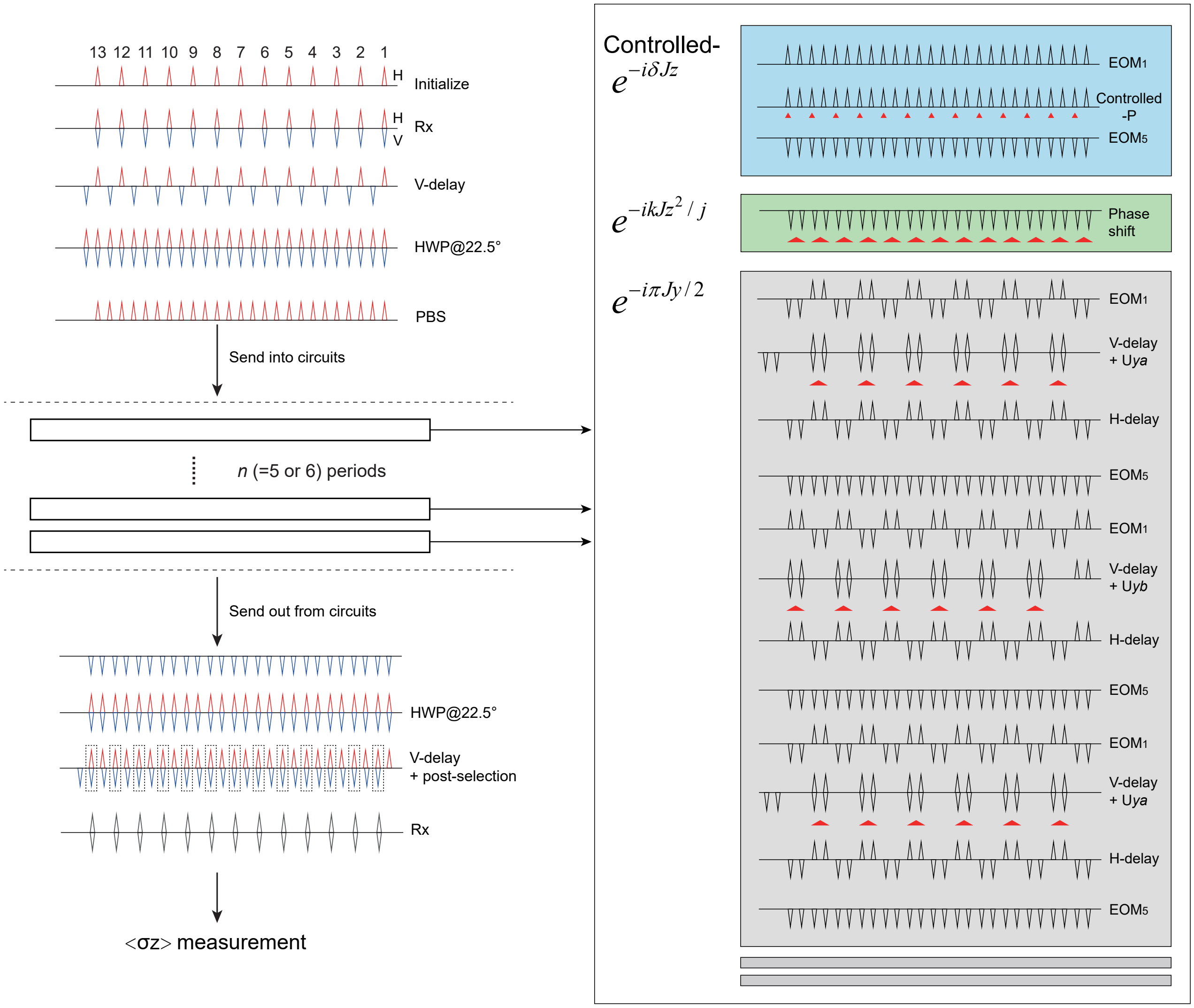

FIG. S5: Pulse diagram during the second program. For each line, the upper pulses represent H polarization, and the lower pulses represent V polarization. The red solid triangles represent the corresponding subevolutions performed by EOM2-4. The second and third gray boxes in the bottom right corner represent two repeats of the first gray box.

offers ultra-low loss, aiding the hardware in displaying high-fidelity sampling results. However, in this work, the use of coherent light from an attenuated laser pulse suffices to demonstrate the features of quantum chaos. This approach is not only due to it being an earlier version of Ref. [4] but also because the coherent light adequately exhibits the quantum chaos characteristics.

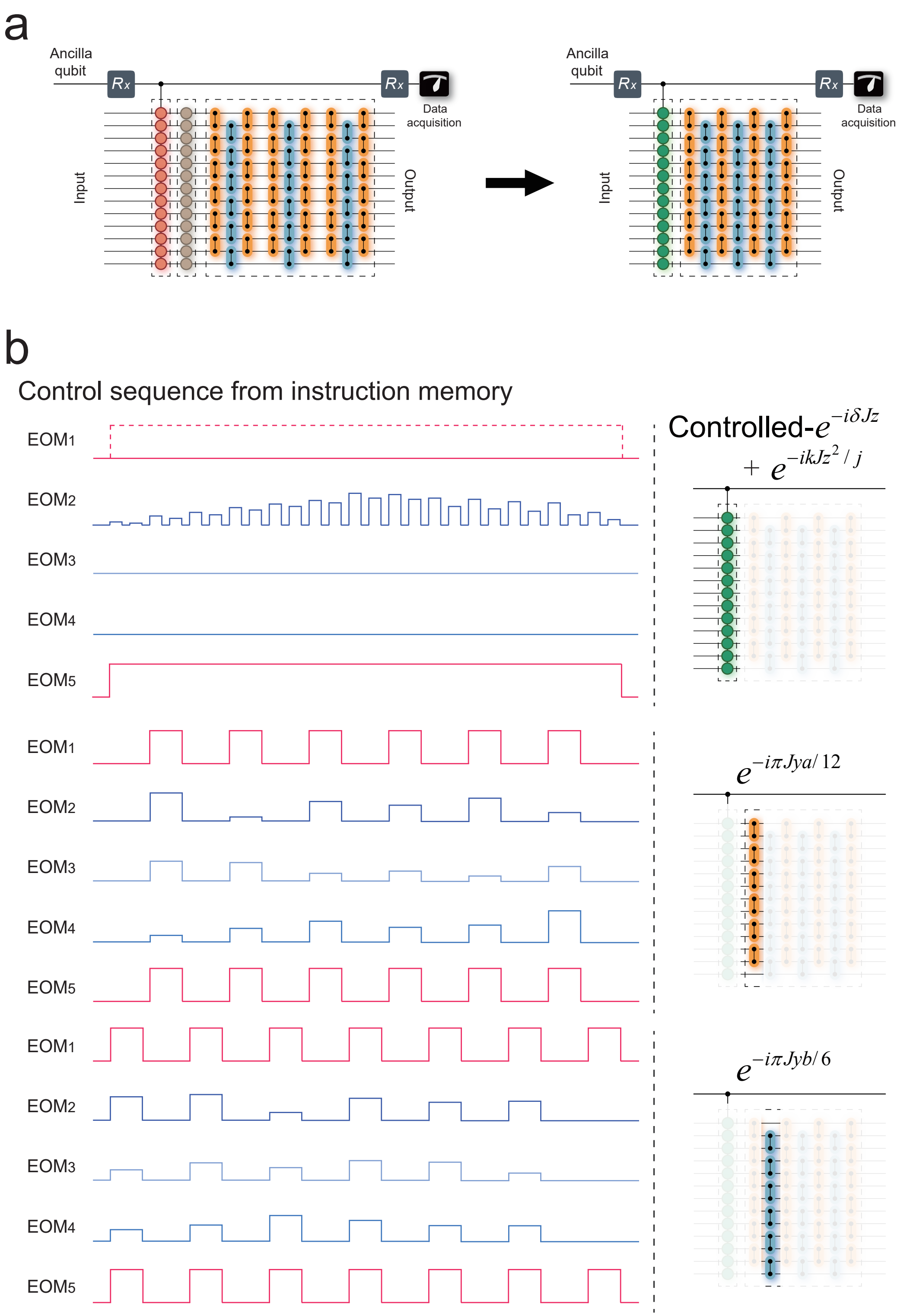
a
Ancilla qubit
Rx
Rx
Data acquisition
Input
Output
Ancilla qubit
Rx
Rx
Data acquisition
Input
Output
b
Control sequence from instruction memory
EOM1
EOM2
EOM3
EOM4
EOM5
Controlled-$e^{-i\delta Jz}$
$+\ e^{-ikJz^2/j}$
EOM1
EOM2
EOM3
EOM4
EOM5
$e^{-i\pi Jya/12}$
EOM1
EOM2
EOM3
EOM4
EOM5
$e^{-i\pi Jyb/6}$

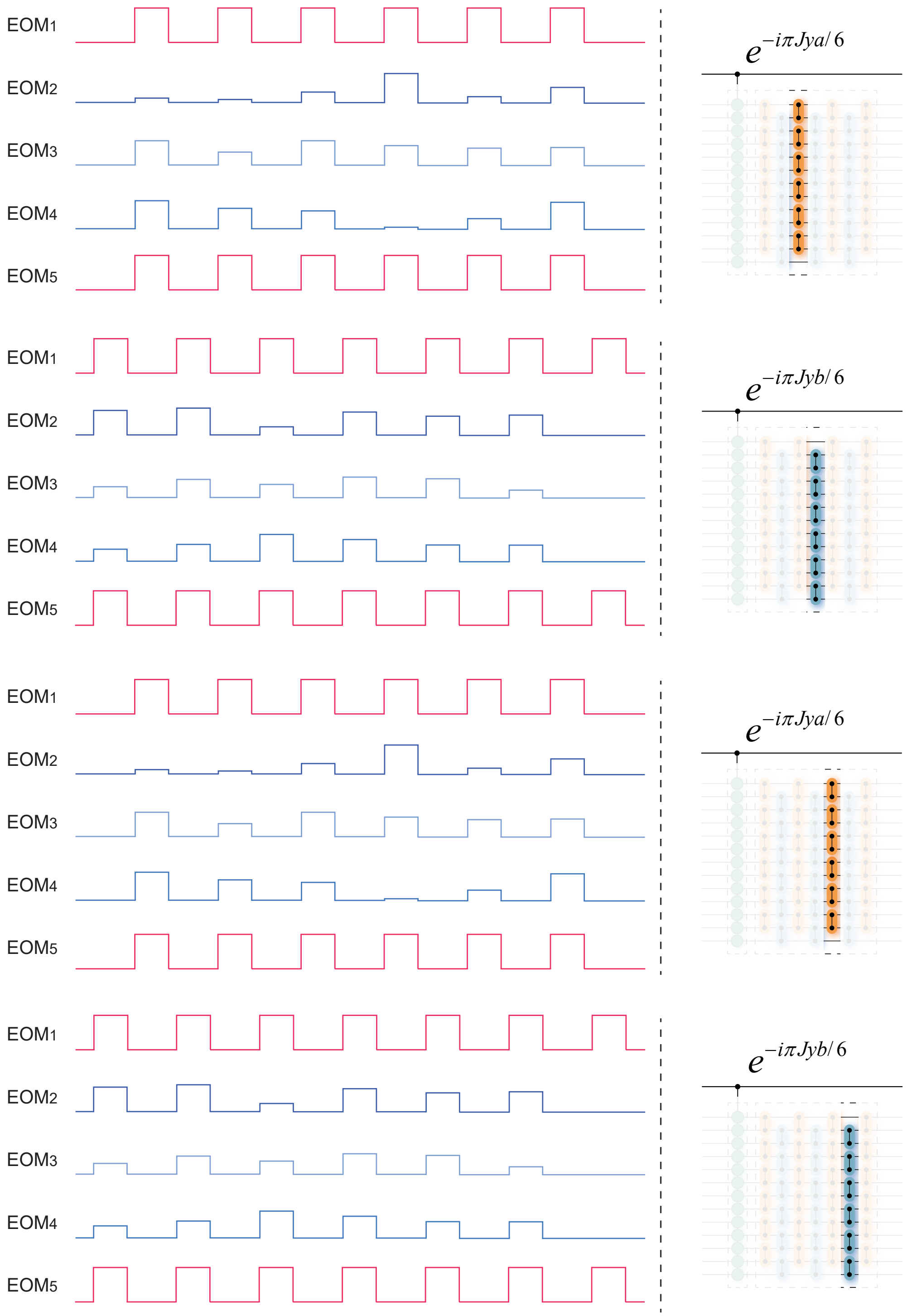
EOM1
EOM2
EOM3
EOM4
EOM5
$e^{-i\pi Jya/6}$
EOM1
EOM2
EOM3
EOM4
EOM5
$e^{-i\pi Jyb/6}$
EOM1
EOM2
EOM3
EOM4
EOM5
$e^{-i\pi Jya/6}$
EOM1
EOM2
EOM3
EOM4
EOM5
$e^{-i\pi Jyb/6}$

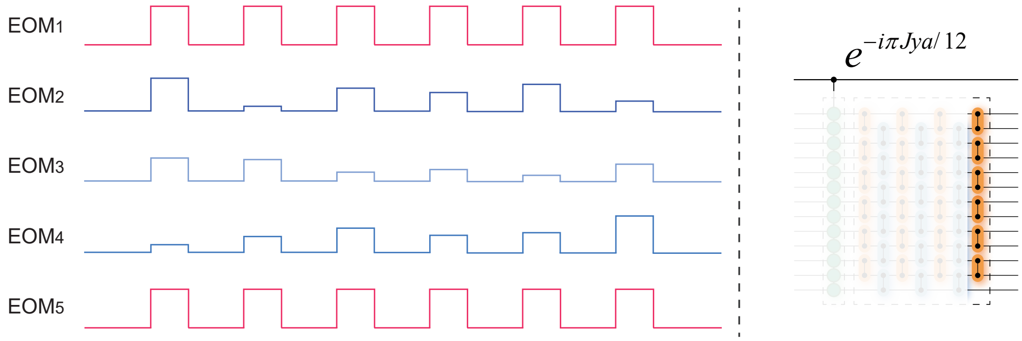

FIG. S6: (a) Combination of loops. (b) Control sequence for one period. We note that this sequence is only a sketch and it has not been practically used in the experiment. Another note is that the control signals for the first line of EOM1 are opposite for the first period (solid line) and the following periods (dashed line), since their input polarization states are opposite.

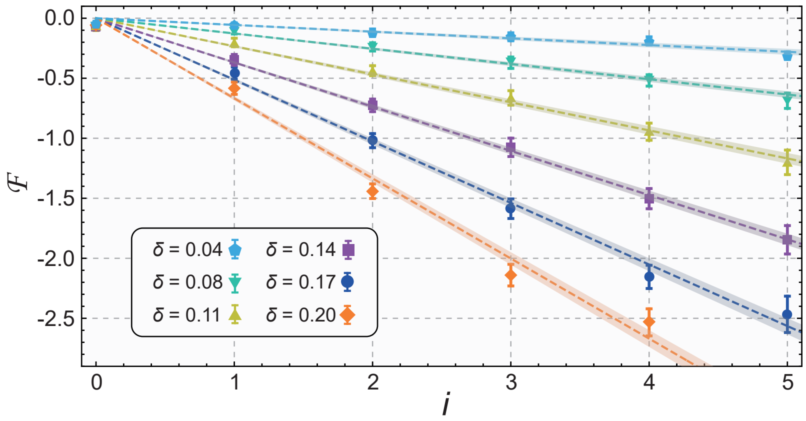

FIG. S7: Fidelity decays with various δ under $k = 12$, and their linear fits. The fitted slopes and corresponding errors constitute Fig. 3c of main text.

XIII. DISCUSSION ABOUT ADVANTAGE OVER CLASSICAL COMPUTERS OR OTHER PHOTONIC PROCESSORS.

We first address the speed advantage over classical computers. Referring to the calculations in Refs. [5, 6], the required number of floating-point operations (FLOPs) per second to match our photonic processor is given by:

$$R = 2m \times N^2 \times f\text{FLOPs} \tag{7}$$

where R represents the number of floating-point operations per second, m denotes the number of iterations in our experiment, N is the number of modes, and f is detection rate for our experiment. Since our experiments utilize a programmable circuit, making f dependent on m, N, and the time gap between adjacent time-bins, i.e., 100 ns. Then, we can calculate that $f = 1\text{s}/((100\text{ns} \times N \times m))$. Consequently, R equates to $2N10^7$ FLOPs, which depends only on the number of modes (the scale of the problem) and the detection rate. It becomes evident that, under a fixed detection rate, the parallelism advantage of optical quantum computation (specifically, the $N \times N$ matrix operation executed at the speed of light on the experimental platform) allows for a linear enhancement in computational power with more modes in our photonic processor.

In the context of this specific quantum chaos problem, we conducted a proof-of-principle validation experiment using the minimal number of modes ($N = 13$) required to exhibit the signature phenomenon of quantum chaos. However, for practical complex systems, the number of modes is often substantial, and this feature highlighting the computational speed advantage of our photonic processor in practical problem. We discovered that with 24,000 modes, our photonic processor could surpass the Intel i7-9700K, which has approximately 460.8 GFLOPs of floating-point

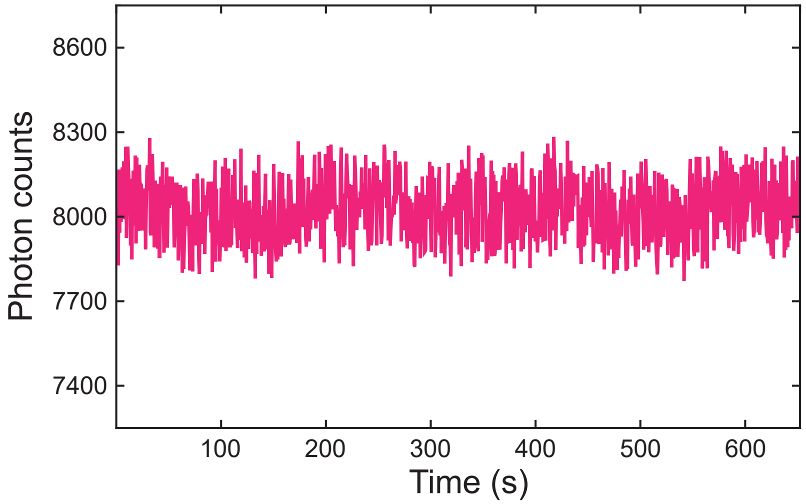

FIG. S8: Photon count fluctuation detected on the $|H\rangle + |V\rangle$ basis after interference between adjacent modes.

computing power.

Moreover, increasing experimental resources by incorporating additional Electro-Optic Modulator (EOM) groups into the setup (multi-core operation shown in Methods and Extended Data Fig. 1) would enable the execution of more matrix multiplication calculations per circulation. In our setup, there is one EOM group (comprising three EOMs, i.e., EOM2-4 in Fig. 1). However, with a combination of Q EOM groups, the FLOPs of our photonic processor would correspondingly increase by a factor of Q. For instance, if the circuit contains eight EOM groups, the photonic processor can then surpass the classical Intel i7-9700K processor, even when tasked with calculating a quantum chaos problem involving only 3000 modes.

Then, we discuss the resource advantages compared to other photonic processors. When contrasted with photonic processors like the integrated (or on-table) photonic chips encoded on spatial modes (typically, path mode), the resource advantages of our time-bin system are clear: it requires only a fixed number of EOMs to solve matrix multiplication problems of any size, facilitating arbitrary scalability without necessitating changes to the experimental setup. Specifically, to perform any $N \times N$ unitary operation, it is typically necessary to prepare $N \times (N-1)$ internal phase shifts and N external phase shifts as separate phase controls on an integrated chip. This demands the characterization and independent control of $N \times N$ shifts. However, with the time-bin encoding method, only the characterization and independent manipulation of 3-5 EOMs are required. Consequently, the necessary experimental resources and complexity of controls are significantly reduced.

Additionally, compared to traditional optical neural networks, our time-bin device is designed towards and can directly accept a quantum light source without requiring any modifications. This capability enables the execution of experiments that feature quantum characteristics, such as photon second-order interference. Consequently, our time-bin device has the potential to provide exponential acceleration and possesses the capability to address many NP-hard problems.

XIV. FURTHER DISCUSSION ABOUT THE PHOTONIC PROCESSOR.

To effectively observe the signature behaviours of quantum chaos in phase space, as well as those related to the Fermi golden rule, it is essential for the hardware to support a large number of modes and possess sufficient circuit depth. Additionally, the hardware needs to be programmable, as various operations are required to explore these two quantum chaos phenomena, especially, the observation of quantum chaos signature and study of Fermi golden rule are two different tasks requiring different resources like mode number and evolution step.

The time-bin setup, known for its strong stability and programmability, is ideally suited for this work. While other systems, such as integrated photonic chips or bulk optical systems, are capable of performing many intriguing quantum simulation tasks, but scaling them up to the necessary scale and depth (especially on the same machine) while simultaneously maintaining programmability is a significant challenge. Especially, the chaos experiment needs many evolution steps, which makes the depth even larger. The time-bin approach, therefore, offers a practical and efficient solution for investigating the complexities of quantum chaos.

Additionally, our memory-inclusive device is highly applicable in optical neural network research. This work has established that any unitary and phase operations can be effectively executed. For coherent light, incorporating nonlinear crystals directly into the circuit enables the completion of necessary nonlinear operations on the nodes within the neural network (similar to the scheme in Ref. [6]).

Besides, this memory-included setup is also particularly adept at quantum simulation, providing enhanced precision and efficiency in simulating quantum behaviours of molecules and materials. In quantum optimization, these processors excel by exploring larger solution spaces for complex problems, potentially outperforming classical computers. The immense parallelism afforded by quantum memory makes them highly effective in quantum machine learning, especially when processing large-scale datasets.

Ultimately, in quantum networking, quantum memory serves as a vital component for storing and relaying quantum information, paving the way for long-distance quantum communication and a quantum internet, and importantly, the distributed quantum computation. Despite these potentials, the field faces challenges like quantum error correction and the need for efficient quantum algorithms, indicating that quantum computing is still in its early developmental stages.

[1] Hsiao, Y.-F. *et al.* Highly Efficient Coherent Optical Memory Based on Electromagnetically Induced Transparency. *Phys. Rev. Lett.* **120**, 183602 (2018).

[2] Agarwal, G. S. Relation between atomic coherent-state representation, state multipoles, and generalized phase-space distributions. *Physical Review A* **24**, 2889-2896 (1981).

[3] Gazeau, J. P. & Klauder, J. R. Coherent states for systems with discrete and continuous spectrum. *Journal of Physics A: Mathematical and General* **32**, 123-132 (1999).

[4] Yu, S. *et al.* A universal programmable Gaussian Boson Sampler for drug discovery. *Nature Computational Science* **3**, 839-848 (2022).

[5] Fu, T. Z. *et al.* Photonic machine learning with on-chip diffractive optics. *Nature Communications* **14**, 70 (2023).

[6] Shen, Y. C. *et al.* Deep learning with coherent nanophotonic circuits. *Nature Photonics* **11**, 441-446 (2017).
